# Effective D-A-D type chromophore of fumaronitrile-core and terminal alkylated bithiophene for solution-processed small molecule organic solar cells

**DOI:** 10.1038/srep11143

**Published:** 2015-06-12

**Authors:** M. Nazim, Sadia Ameen, Hyung-Kee Seo, Hyung Shik Shin

**Affiliations:** 1 Energy Materials & Surface Science Laboratory, Solar Energy Research Center, School of Chemical Engineering, Chonbuk National University, Jeonju 561-756, Republic of Korea

## Abstract

A new and novel organic π-conjugated chromophore (named as RCNR) based on fumaronitrile-core acceptor and terminal alkylated bithiophene was designed, synthesized and utilized as an electron-donor material for the solution-processed fabrication of bulk-heterojunction (BHJ) small molecule organic solar cells (SMOSCs). The synthesized organic chromophore exhibited a broad absorption peak near green region and strong emission peak due to the presence of strong electron-withdrawing nature of two nitrile (–CN) groups of fumaronitrile acceptor. The highest occupied molecular orbital (HOMO) energy level of –5.82 eV and the lowest unoccupied molecular orbital (LUMO) energy level of –3.54 eV were estimated for RCNR due to the strong electron-accepting tendency of –CN groups. The fabricated SMOSC devices with RCNR:PC_60_BM (1:3, w/w) active layer exhibited the reasonable power conversion efficiency (PCE) of ~2.69% with high short-circuit current density (J_SC_) of ~9.68 mA/cm^2^ and open circuit voltage (V_OC_) of ~0.79 V.

In organic solar cells (OSCs), organic π-conjugated chromophores have shown a great potential as an alternative to organic π-conjugated polymers in solution-processed bulk-heterojunction (BHJ) organic photovoltaic devices due to their various advantages such as light weight, flexibility, low cost, and the ease of synthesis and fabrication-processing^1^,^2^,^3^,^4^,^5^. Organic chromophores with aromatic fumaronitrile-core have attracted a significant attention in electroluminescent (EL) devices due to their efficient emission properties in the solid state^6^,^7^,^8^. The presence of diphenylfumaronitrile-core greatly reduces the fluorescence quenching in the solid state because of the interaction of anti-parallel dipoles^9^,^10^. In last decade, a lot of substantial efforts have been performed for improving the device performances of solution-processed small molecule organic solar cells (SMOSCs) and attained the high PCE through the development of organic photoactive elecctron-donor materials^11^,^12^. The achievement of PCEs of over ~8% in SMOSCs has made them a serious candidates for the next generation of solar cells, polymer solar cells (PSCs), thin film solar cells and dye-sensitized solar cells (DSSCs)^13^,^14^. The high-efficiency solar cell devices have been reported for a solution-processed bulk-heterojunction (BHJ) OSCs containing low-band gap semiconducting polymers and [6,6]-phenyl-C_61_-butyric acid methyl ester (PC_61_BM), or [6,6]-phenyl-C_71_-butyric acid methyl ester (PC_71_BM)^15^,^16^.

Oligothiophenes have been employed as organic electron-donors owing to their high electron-density, well-defined and planar structure, and good solubility for the design and construction of optical and organic electronic materials. The development of organic conjugated donor–acceptor–donor (D–A–D) systems composed of oligothiophenes (donor) and electron-deficient molecule (acceptor) provides an efficient approach since these molecules could increase an absorption band through intramolecular charge transfer (ICT) for better match-up of the solar spectrum and thus, attain the high PCE in organic solar cells devices^17^,^18^,^19^.

In general, the presence of –CN group in organic polymers lowers HOMO and LUMO values compared to without –CN-groups analogues^20^,^21^,^22^,^23^,^24^,^25^,^26^. However, only a few –CN group-modified polymers have been reported to function in photovoltaic devices^27^. To achieve organic compounds with –CN group for PSCs is still a challenge and more efforts are needed to explore the fundamental aspect of –CN-group based D−A systems for high performance devices^28^. In this regard, a new, symmetrical D–A–D organic semiconductor framework is designed with the fumaronitrile (FN) as an electron-withdrawing moiety and utilized in solution-processed SMOSCs. In this study, we report the synthesis and organic photovoltaic characteristics of a novel and efficient D–A–D type (Fig. 1) fumaronitrile-based organic π-conjugated chromophore, 2,3-bis(4-(5-(5-hexylthiophen-2-yl)thiophen-2-yl)phenyl) fumaronitrile (named as RCNR). The photovoltaic characteristics are significantly influenced by the self-assembly behaviour of RCNR due to the existence of different liquid-crystal phases.

## Results and Discussion

**Figure 2:**
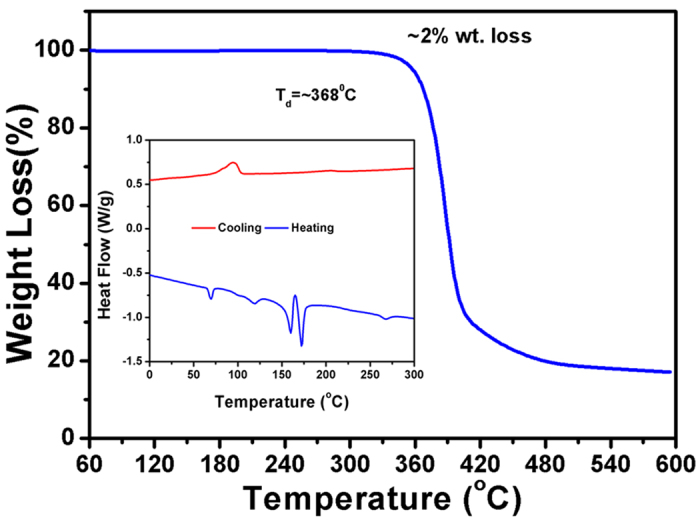
Thermogravimetric analysis (TGA) and Differential scanning colorimetry (DSC) plots of the organic chromophore.

The thermal stability of the synthesized organic chromophore, RCNR has been analysed by the thermogravimetric analysis (TGA) and differential scanning calorimetry (DSC) under N_2_ atmosphere. The TGA plot (Fig. 2) reveals that RCNR starts to decompose over ~300 °C. The decomposition temperature (T_d_) of the RCNR is found as ~368 °C, indicating a relatively high thermal-stability of the organic chromophore which is expedient for the solution-processed device fabrication and the operation of organic solar cells^29^. From differential scanning calorimetry (DSC) measurement (Fig. 2 inset), the RCNR show numbers of melting phase transitions (T_m_) at ~69 °C, ~161 °C, and ~172 °C, with no signs of a glass-transition temperature (T_g_), while an isotropic transition phase is observed after ~270 °C^30^. The increase in the thermal transition temperatures is an indication of enhanced intermolecular connectivity and thin film crystallinity in RCNR, which is attributed to the presence of induced π−π stacking^31^. The difference in film crystallinity is an important factor for solution-processed organic solar cells, as it shows a direct effect on the surface roughness of the thin film morphology and consequently, the solar cell device performance^32^. The presence of terminal alkyl chains of organic chromophore induces the solubility in common organic solvents. Additionally, different melting transitions suggest the occurrence of various liquid-crystalline (LC) phases of RCNR via self-assembly behavior^33^. Generally, self-assembly behavior is the result of electrostatic interactions which might be due to the result of π−π staking and hydrogen-bonding ability of the organic conjugated molecules^34^,^35^. This clearly indicates the interconversion of different LC phases from smectic C to smectic A to nematic phase as a function of temperature^36^,^37^.

**Figure 3:**
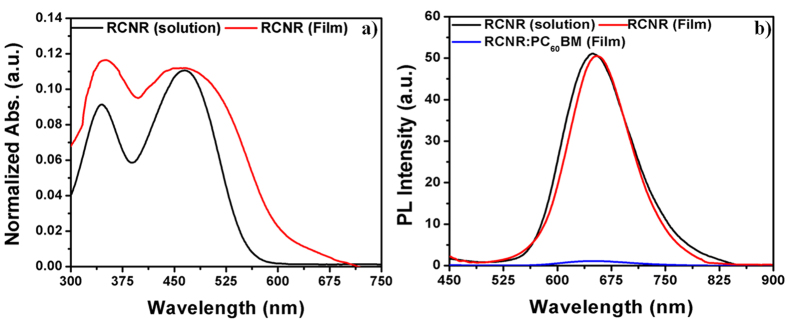
(**a**) Ultraviolet-visible (UV-Vis) spectra of RCNR in chloroform solution (Black line) and thin film (Red line) deposited on ITO substrate, and (**b**) Photoluminescence spectra of RCNR in chloroform solution (Black line), thin film (Red line) and RCNR:PC_60_BM (1:3, w/w) active layer thin film (Blue line).

UV-Vis absorption spectra (Fig. 3a) of RCNR have displayed a good absorption in dilute chloroform solution (1 × 10^−5^ M) and thin film state. In chloroform solution, the two distinct peaks are observed. The spectra shows a relatively small absorption peak at λ_max_ ≈ 368 nm and another broad absorption peak at λ_max_ ≈ 465 nm. The molar absorption coefficient (ε) in solution is calculated as ~1.58 × 10^4^ M^−1^ cm^−1^ which indicates a strong intramolecular charge transfer (ICT) interaction behavior between thiophene donor and fumaronitrile-acceptor^38^,^39^. However, a slight red shift with broad absorption spectrum (Table 1) is observed for the chromophore in the solid thin film as compared to chloroform solution which might be due to an aggregation in the solid thin film state^40^. RCNR indicates an ordered and planar structure due to the presence of alkyl side chains, resulting in a good intermolecular electron-delocalization and hence, evolves the self-assembly behavior^41^,^42^. Moreover, an optical band gap (E_g_^opt^) of ~2.03 eV is calculated by the absorption edge (λ_edge_) from solid thin film absorption by the formula:


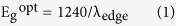


The photoluminescensce spectra (Fig. 3b) of the synthesized organic chromophore has shown a good potential of light emitting properties in solution as well as solid thin film state. A single storng green emission peak at ~649 nm is recorded in chloroform solution at the room temperature which shows a slight red-shift in thin film spectra. This strong emission of RCNR is due to the intramolecular planarization or aggregation of organic chromophore^43^. It clearly indicates the fluorescence quenching after mixing with PC_60_BM acceptor, suggesting the electron transfer from donor to acceptor and the fast charge-transfer which is enough to compete with the radiative recombination of the excitons^44^,^45^.

**Figure 4:**
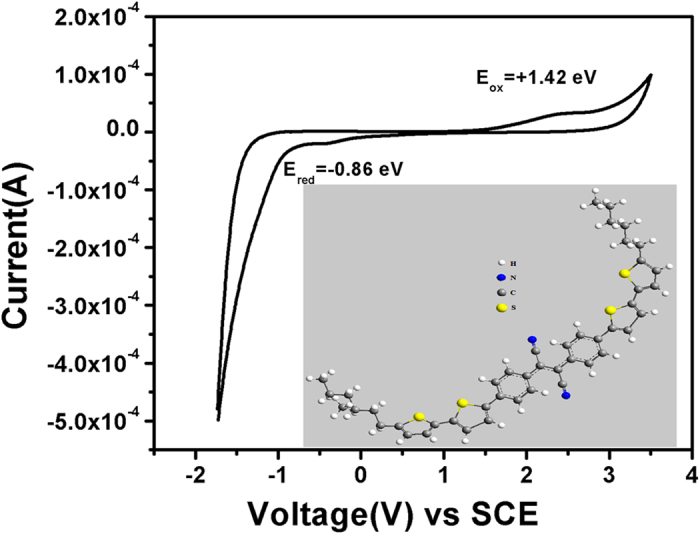
Cyclic Voltammogram of RCNR thin film in 0.1 M acetonitrile solution containing [^n^Bu_4_N]^+^[PF_6_]^−^ as supporting electrolyte with a scan rate of 100 mV/s.

The redox properties of the organic chromophore are measured by cyclic voltammetry (CV) studies of RCNR thin film (Fig. 4) in 0.1 M CH_3_CN solution of tetrabutyl ammonium hexa flouro phosphate [^n^Bu_4_N]^+^[PF_6_]^−^ at a potential scan rate of 100 mV/s. The oxidation and reduction peaks are situated at the onset value of E_ox_ = +1.42 ± 0.02 eV and E_red_ = –0.86 ± 0.02 eV. Hence, the RCNR solid thin film exhibits HOMO and LUMO of –5.82 eV and –3.54 eV, respectively. The observed electrochemical band gap is found to be E_g_^el^ = 2.28 eV. The difference of HOMO and LUMO energy level is a crucial factor for determining the energy band gap which indicates the electrons delocalization in the solid thin films^46^,^47^.

**Figure 5:**
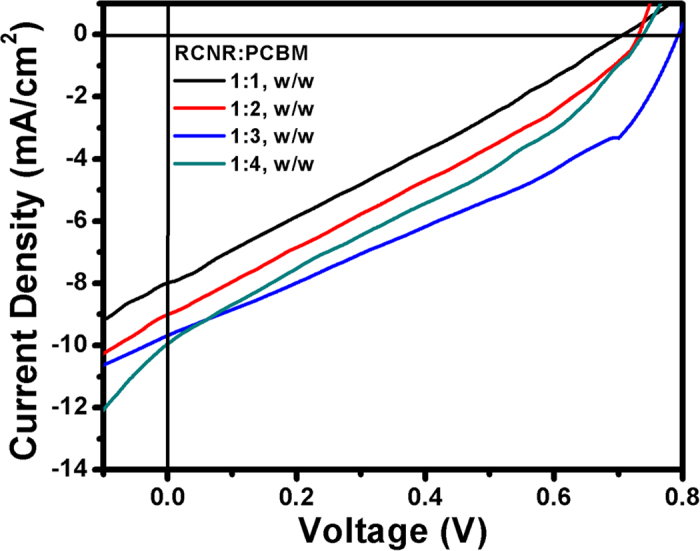
J-V curves of fabricated small molecule organic solar cells with the different RCNR:PC_60_BM active layers.

Solution-processed BHJ small molecule organic solar cells are fabricated using RCNR as an electron-donor and [6,6]-phenyl C_61_-butyric acid methyl ester (PC_60_BM) as an electron-acceptor with a standard device structure of ITO/PEDOT:PSS (~80 nm)/RCNR:PC_60_BM blend (~60 nm)/Ag (~100 nm). The blended active layers of the solar cell devices are developed by spin-casting the various (1:1, 1:2, 1:3, 1:4, w/w) mixtures of the RCNR with PC_60_BM. The photovoltaic properties (Table 2) of the fabricated solar cell devices of RCNR have been examined by the current density (J)-voltage (V) measurements (Fig. 5) under the 1 sun light (100 mW/cm^2^, 1.5 AM). The PCE of ~2.69% is achieved by the SMOSC devices fabricated with RCNR:PC_60_BM (1:3, w/w) active layer ratio, whereas the other fabricated SMOSC devices exhibit inferior PCEs of ~1.50% for RCNR:PC_60_BM (1:1, w/w), ~2.0% for RCNR:PC_60_BM (1:2, w/w) and ~2.23% for RCNR:PC_60_BM (1:4, w/w) active layer ratios. The SMOSC fabricated with RCNR:PC_60_BM (1:3, w/w) active layer presents the J_SC_ of ~9.68 mA/cm^2^, and high V_OC_ of ~0.792 V. Herein, the presence of –CN groups connecting with vinyl double bond enhances the conjugation length of chromophore and hence, better electron-delocalization which might affect the open-circuit voltage and short-circuit density of the solar cell devices^8^,^48^. Moreover, the presence of the terminal side chains has a strong impact on the aggregation and self-organizing behavior of the electron-donor molecules in BHJ thin films and hence, increases the photocurrent-density of the devices due to better charge transport^49^. The thin film morphology of the devices might be related to the lowering of the V_OC_ value at low concentration ratios (1:1, 1:2, w/w) of RCNR in the blended active layers.

**Figure 6:**
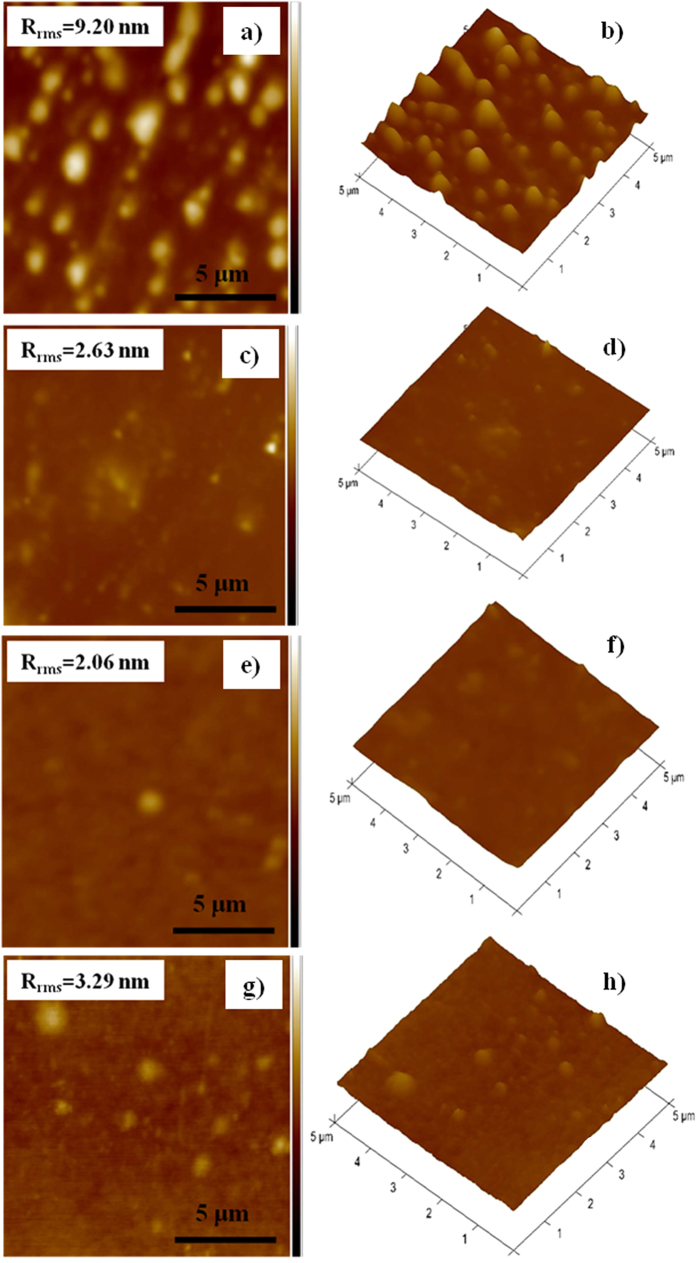
Topographic and three dimentional AFM images of the fabricated small molecule organic solar cells device of various ratios with RCNR:PC_60_BM (**a**,**b**) 1:1 w/w, (**c**,**d**) 1:2 w/w, (**e**,**f**) 1:3 w/w and (**g**,**h**) 1:4 w/w active layers.

The atomic force microscopy (AFM) analysis is used to investigate the morphological behavior of the blended active layer RCNR:PC_60_BM (1:1, 1:2, 1:3, 1:4, w/w) thin films, as shown in Fig. 6. The RCNR:PC_60_BM (1:3, w/w) blended active layer (Fig. 6(e,f)) clearly exhibits a homogeneous and smooth morphology of low root-mean-square surface roughness (R_rms_ = 2.06 nm) in nanoscale phase separation which contributes to good miscibility of donor-acceptor, high exciton-dissociation rate and better charge transport. On the other hand, other blended active layers of RCNR:PC_60_BM (1:1, 1:2 and 1:4, w/w) record high R_rms_ values of 9.20 nm, 2.63 nm, 3.29 nm, respectively. These results show that the RCNR:PC_60_BM (1:3, w/w) active layer is the optimized one for homogeneous miscibility between donor and acceptor yielding a smooth thin film morphology and a large donor-acceptor (D−A) interface area with nanoscale phase separation^33^ and high exciton-dissociation rate, which eventually assists to achieve the best performance of organic solar cell devices. In addition, the morphological analysis reveals that RCNR:PC_60_BM (1:3, w/w) film depicts the lowest R_rms_ of ~2.06 nm as compared to other blended RCNR:PC_60_BM (1:1, 1:2 and 1:4, w/w) active layers, suggesting the homogenous nature of RCNR and PC_60_BM molecules in the blended layer which provides enough surface area for exciton-dissociation^40^. For all the fabricated SMOSCs devices, the fill factor (FF) value is rather low due to a number of factors like unfavourable domain size, film morphology, misalignment of energy levels, large series-resistance, etc^44^. The active layer RCNR:PC_60_BM (1:3, w/w) device shows a minimum series-resistance and hence, a maximum FF of ~0.35. Furthermore, the lower values of FF are related to increase in the series-resistance of RCNR:PC_60_BM/ITO in SMOSCs, resulting in a higher recombination rate over the surface of RCNR:PC_60_BM blended active layers^6^. Due to spontaneous phase-segregation process in the blended active layers of RCNR and PC_60_BM, a bicontinuous network structure might form which creates the percolation channels for the efficient charge carrier collection within the active layer of BHJ solar cells^50^. On the other hand, the improvement in the V_OC_ value might be due to the presence of two –CN groups which induce the better film morphology and strong intermolecular charge-transfer (ICT) between RCNR and PC_60_BM^9^,^51^. Thus, the presence of two strong electron-withdrawing –CN groups might have electrostatic-attractions with PC_60_BM which improves the film-morphology of the blended active layers and ultimately increases the photocurrent-density for the better performance of solar cell devices^52^.

## Experimental Methods

### Instruments

Unless otherwise noted, the chemicals and reagents were purchased from commercial sources as Sigma–Aldrich, Alfa-aesar and TCI chemical companies and used as received. Thin layer chromatography (TLC) was performed on Merck TLC-plates of aluminum coated with silica gel 60 F254. Flash column chromatography was performed on a column packed with silica gel (300–400 mesh). Fourier transform-infrared (FTIR) spectroscopy was performed by FT/IR-4100 (JASCO) spectrometer. Ultra violet–visible (UV-vis) absorption and photoluminescence spectra (PL) were recorded by V-670 (JASCO) spectrophotometer and FP-6500 (JASCO) fluorometer, respectively. The cyclic voltammetry (CV) measurements were done using WPG 100 Potentiostat/Galvanostat (WonATech) at a scan rate of 50 mV/s with a three-electrode cell consisting of a glassy carbon working electrode, a saturated calomel reference electrode (SCE) and a platinum wire as counter electrode. CV measurement was performed in 0.1 M solution of tetrabutylammonium hexafluorophosphate (TBAPF_6_) in acetonitrile as the supporting electrolyte. The RCNR was dissolved in chloroform solvent and thin film was deposited on the glassy carbon working electrode by drop casting and dried at 60 °C for 4 h under nitrogen. NMR spectra were obtained in CDCl_3_ solvent (^1^H at 600 MHz and ^13^C at 100 MHz) using JEOL FT-NMR spectrophotometer. For NMR analysis, CDCl_3_ was used as the solvent and chemical shift values (*δ* values) were considered in parts per million (ppm) with tetramethylsilane (TMS) as the internal reference. Thermo-gravimetric analysis (TGA) was carried out with a TA instruments Q-50 thermogravimetry analyzer at a scan rate of 10 °C/min under inert atmosphere. The differential scanning calorimetry (DSC) was characterized by TA instrument DSC-2910 at heating rate of 10 °C/min under nitrogen atmosphere.

### Synthesis

The reaction intermediates **2, 3, 4, 5,** and **7** were synthesized as reported elsewhere^6^,^44^,. In brief, the target product, RCNR was finally obtained by the Suzuki cross-coupling reaction between intermediate **7** and intermediate **5** using Pd(PPh_3_)_4_ (2.5 mol %) as catalyst and potassium carbonate, K_2_CO_3_ as a base in anhydrous toluene solvent under inert atmosphere. The synthesized red-colored chromophore was then purified by repeated crystallization in the mixed solvent of dichloromethane/methanol (2:1, v/v) with a decent yield of 86.4%.

### 1-(5-(thiophen-2-yl) thiophen-2-yl) hexan-1-one (2)

Hexanoyl chloride (4.07 mL, 20.0 mmol) was added to a solution of 2,2’-bithiophene **1** (3.17 g, 19.1 mmol) in anhydrous benzene (20 mL) at the room temperature. Then TiCl_4_ (2.25 mL, 20.5 mmol) was added slowly to the reaction mixture at 0°C and was stirred for 15 min at 0 °C. After completion of the reaction, cold water was added into the reaction mixture to quench the reaction. The resulting mixture was diluted with CH_2_Cl_2_ (50 mL), washed successively with water (200 mL) and saturated aqueous solution of NaHCO_3_ (100 mL), then dried over MgSO_4_ followed by an evaporation under vacuum to afford a yellow solid (5.00 g, 85%), anticipated as the desired ketone intermediate **2** which was used directly for next step of the reaction.

### 2-Decyl-5-(thiophen-2-yl) thiophene (3)

Under nitrogen atmosphere, the solution of intermediate **2** (5.00 g, 18.9 mmol) in anhydrous toluene (40 mL) and a suspension of LiAlH_4_ (4.6 g, 121 mmol) and AlCl_3_ (4.03 g, 30.3 mmol) in anhydrous Et_2_O (100 mL) were mixed slowly at 0 °C with extreme care. The reaction mixture was then stirred for 1 h at the room temperature, and again cooled at 0 °C, then ethyl acetate (20 mL) and HCl (6 M) solution (50 mL) were added to the reaction mixture. The resulting mixture was extracted with diethyl ether (300 mL), washed with NaCl solution and distilled water (50 mL) thereafter, dried over MgSO_4_. Afterward, solvent of the organic phase was evaporated by using rotatory evaporator followed by drying in the vacuum. The yellow residue was then purified by flash column chromatography on silica gel (hexane) to produce compound **3** (4.65 g, 98%) as a colourless oil. ^1^H NMR (400 MHz, CDCl_3_, δ, ppm): 7.15 (d, 1H), 7.10 (d, 1H), 6.96 (d, 1H), 6.95 (d, 1H), 6.65 (d, 1H), 2.75 (t, 2H), 1.69 (m, 2H), 1.35 (m, 14H), 0.90 (t, 3H); ^13^C NMR (100 MHz, CDCl_3_, δ, ppm): 146.4, 145.2, 138.0, 134.8, 127.7, 125.7, 124.3, 123.8, 123.5, 123.0, 32.0, 31.7, 30.2, 29.5, 29.4, 29.2, 22.8, 14.2.

### 5-Bromo-5’-decyl-2,2’-bithiophene (4)

N-bromo succinimide (1.22 g, 6.86 mmol) was added to a solution of compound **3** (2.00 g, 8.0 mmol) in dimethylformamide (30 mL) and the reaction mixture was stirred for 30 min in the absence of light then, diluted with hexane (50 mL), washed with saturated aqueous solution of NH_4_Cl (50 mL), dried over MgSO_4_, and evaporated under vacuum. The residue was purified by column chromatography on silica gel (hexane) to give a white solid compound **4** (2.36 g, 89.9%). ^1^H NMR (400 MHz, CDCl_3_, δ, ppm): 6.95 (d, 1H), 6.90 (d, 1H), 6.85 (d, 1H), 6.68 (d, 1H), 2.78 (t, 2H), 1.66 (m, 2H), 1.35 (m, 14H), 0.90 (t, 3H); ^13^C NMR (100 MHz, CDCl_3_, δ, ppm): 145.6, 139.9, 133.5, 130.5, 130.1, 124.8, 123.7, 123.1, 110.2, 32.2, 31.7, 30.3, 29.9, 29.6, 29.4, 22.9, 14.4.

### 2-{5-(5-Decylthiophen-2-yl) thiophen-2-yl}-4,4,5,5-tetramethyl-1,3,2-dioxaborolane (5)

Under nitrogen atmosphere, a solution of compound **4** (1.0 g, 3.06 mmol) and tetrahydrofuran (20 mL) was added to n-BuLi (1.6 M, 3.17 mmol) at –78 °C. The temperature was increased slowly up to –50 °C within 20 min. 2-Isopropoxy-4,4,5,5-tetramethyl-1,3,2-dioxaborolane (0.58 mL, 5.27 mmol) was added and the temperature was increased slowly to the room temperature. The reaction mixture was then stirred for 3 h at room temperature and 2N HCl (20 mL) was added. The resulting mixture was extracted with diethylether (30 mL), washed with NaCl solution followed by distilled water (500 mL), dried over MgSO_4_, and evaporated under vacuum. The obtained residue was recrystallized from hexane (10 mL) to yield a white solid compound, **5** (0.85 g, 73.3%). ^1^H NMR (400 MHz, CDCl_3_, δ, ppm): 7.55 (d, 1H), 7.20 (d, 1H), 7.05 (d, 1H), 6.70 (d, 1H), 2.81 (t, 2H), 1.72 (m, 2H), 1.38 (m, 14H), 0.91 (t, 3H); ^13^C NMR (100 MHz, CDCl_3_, δ, ppm): 146.1, 144.8, 137.9, 134.7, 124.9, 124.5, 124.1, 84.1, 31.9, 31.5, 30.2, 29.6, 29.4, 29.3, 24.8, 22.6, 14.3.

### Bis (4-bromophenyl) fumaronitrile (7)

A mixture of 4-bromophenylacetonitrile, **6** (4.86 g, 24.8 mmol) and iodine (6.35 g, 25 mmol) was purged with N_2_ and subsequently anhydrous diethyl ether (100 ml) was injected via syringe. A solution was cooled to –78 °C. Sodium methoxide (NaOCH_3_, 2.84 g, 52.6 mmol) and methanol (40 ml) was added slowly over a period of 30 min and then stirred for 40 min. Then the reaction solution was put to ice-water bath at 0 °C with stirring for further 4 h. Hydrochloric acid (3–6%) was added dropwise to the reaction mixture and the solution was filtered to isolate the precipitate, which was then rinsed with cold methanol-water solution. Filtrate was concentrated further and a second crop of target product was obtained as a pale yellow solid, **7** (5.87 g, 61.3%). FT-IR (KBr pellet, cm^−1^): 3096, 2220, 1585, 1488, 1396, 1245, 1074, 1007, 845, 816, 710, 665, 627, 573, 514. ^1^H NMR (400 MHz, CDCl_3_, δ, ppm):7.67-7.72 (m, 8H).

### 2,3-bis(4-(5-(5-hexylthiophen-2-yl)thiophen-2-yl)phenyl)fumaronitrile (RCNR)

In a 50 mL round bottom flask, monomer **5** (0.46 g, 1.22 mmol) and monomer **7** (0.198 g, 0.51 mmol) with triphenylphosphine (0.034 g, 0.03 mmol) were mixed and then subjected to three cycles of evacuation and nitrogen purging in anhydrous toluene (~10 mL) solvent. Aqueous solution of potassium carbonate (2 M, ~5 mL) was added by syringe to the reaction mixture and was stirred at 110 °C for 12 h. The reaction mixture was cooled down to the room temperature followed by the addition of water. Subsequently, an organic phase was extracted with dichloromethane (~20 mL) and the reaction mixture was washed with brine and distilled water and dried over magnesium sulfate. The solution was filtered and evaporated in vacuum to achieve a red colored residue, which was then recrystallized several times in dichloromethane and methanol (2:1 v/v) mixture to get organic chromophore as a dark red solid (0.32 g, 86.4%). FT-IR (KBr pellet, cm-1): 3067, 2955, 2926, 2853, 2219, 1631, 1581, 1488, 1396, 1245, 1084, 1007, 845, 816, 710, 665, 627. ^1^H NMR (600 MHz, CDCl_3_, δ, ppm): 7.89–7.87 (d, 4H, Ar H), 7.75-7.71 (d, 4H, ArH), 7.55–7.52 (d, 2H), 7.36-7.34 (d, 2H) 7.11-7.02 (d, 2H), 6.72-6.71 (d, 2H), 2.84-2.78 (d, 4H), 1.70-1.68 (m, 4H), 1.39-1.31 (m, 12H), 0.92-0.88 (d, 6H). ^13^C NMR (100 MHz, CDCl_3_, δ, ppm): 145.2, 139.2, 138.2, 136.3, 133.2, 129.1, 128.6, 128.4, 124.8, 124.6, 124.1, 122.9, 116.2, 115.9, 30.6, 30.4, 29.2, 29.0, 27.8, 27.5, 21.5, 21.3, 12.9; MS: m/z 726 (M^+^).

### Device fabrication

For the fabrication of SMOSCs, the indium tin oxide (ITO) glass substrate was first cleaned with detergent, ultrasonicated in water, acetone and isopropyl alcohol and subsequently dried overnight in an oven. PEDOT:PSS thin film (thickness ~80 nm) was coated on ITO substrates by spin-coating the solution with a speed of ~4000 rpm for 40 s and thereafter, annealed at 130 °C for 10 min in a vacuum oven. The active RCNR:PC_60_BM layer (thickness ~60 nm) with w/w blending ratio of 1:1, 1:2, 1:3 or 1:4 in o-dichlorobenzene solution (10 mg/ml) was again spin-coated on PEDOT:PSS film-coated ITO at a scan rate of ~700 rpm for 40 s. The fabricated active layer was heated at 80 °C for 10 min to evaporate the residual solvent. Finally, the silver cathode (thickness ~100 nm) was deposited through a shadow mask by thermal evaporation under a vacuum of about 3 × 10^–6^ Torr. The active area of device was measured as ~1.5 cm^2^. The photovoltaic properties of the cells were measured under simulated AM 1.5 radiation at 100 mW/cm^2^ using 1000 W metal halide lamp (Phillips) which was served as a simulated sun light source and its light intensity (or radiant power) was adjusted with a Si photo detector fitted with a KG-5 filter (Schott) as a reference, calibrated at NREL (USA). The power conversion efficiency (*η*) is calculated by the following equation:


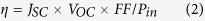


where J_SC_ is the short-circuit photocurrent density, V_OC_ is the open-circuit voltage, FF is the fill factor, and P_in_ is the incident radiation power.

## Conclusions

A novel, symmetric D-A-D type fumaronitrile-acceptor based organic π-conjugated chromophore (RCNR) is synthesized and applied as an electron-donor material for the solution-processed fabrication of SMOSCs. The synthesized organic chromophore presents a broad absorption peak near green region and strong emission peak due to the presence of two strong electron-withdrawing −CN groups. The cyclic voltammetry study of RCNR shows relatively deep HOMO of −5.82 eV and LUMO of −3.54 eV, which suggests a strong electron-accepting tendency of –CN groups. The fabricated SMOSC device of active layer RCNR:PC_60_BM (1:3, w/w) achieves a reasonable PCE of ~2.69% with J_SC_ of ~9.68 mA/cm^2^ and V_OC_ of ~0.79 V. The variation in the concentration of PC_60_BM acceptor in blended active layers has considerably affected the thin film morphology and hence, the performance of the fabricated solar devices.

## Additional Information

**How to cite this article**: Nazim, M. *et al*. Effective D-A-D type chromophore of fumaronitrile-core and terminal alkylated bithiophene for solution-processed small molecule organic solar cells. *Sci. Rep*. **5**, 11143; doi: 10.1038/srep11143 (2015).

## Figures and Tables

**Figure 1:**
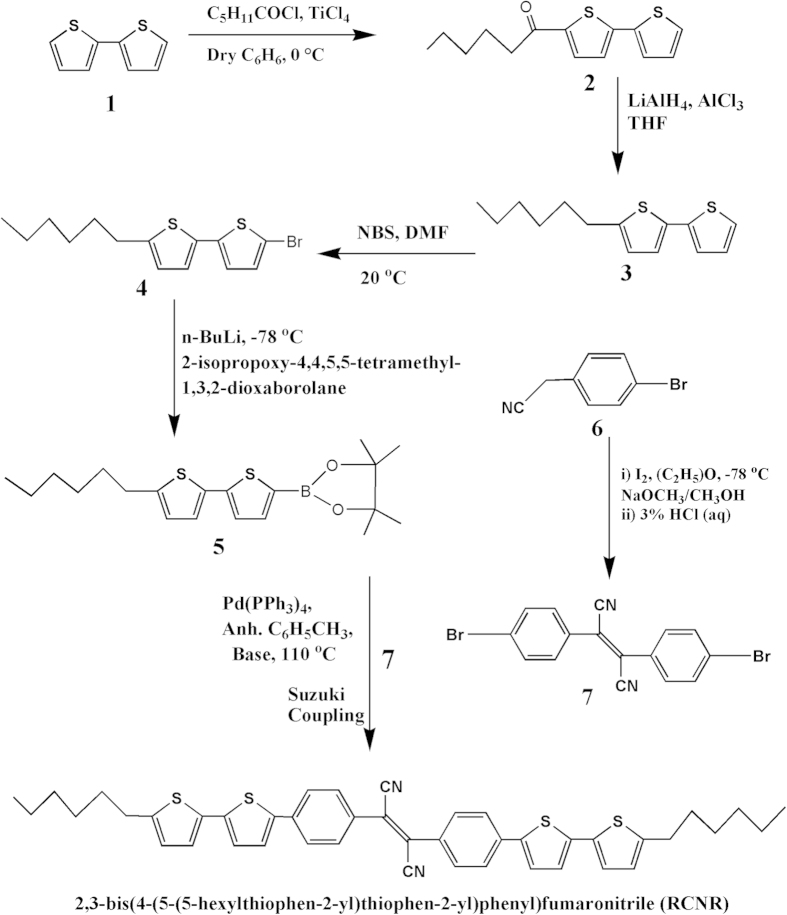
Synthetic route of fumaronitrile based organic chromophore (RCNR).

**Table 1:** Optical and Electrochemical properties of RCNR.

Chromophore	λ_max_^a^ (nm)	λ_max_^b^ (nm)	HOMO^c^ (eV)	LUMO^d^ (eV)	E_g_^e^ (eV)	E_g_^f^ (eV)
**RCNR**	368,465	370,466	−5.82	−3.54	2.28	2.03

^a^Absorption in chloroform solution

^b^Absorption of thin film on ITO

^c^Estimated from the onset of oxidation wave of cyclic voltammogram,

^d^Estimated from the onset of reduction wave of cyclic voltammogram,

^e^Electrochemical band gap calculated from cyclic voltammogram,

^f^Optical band gap calculated from the onset of the UV-vis spectra of the thin film.

**Table 2:** **Summary of J-V curves of the fabricated SMOSCs.**

RCNR:PCBM	Photovoltaic parameters			
	J_SC_ (mA/cm^2^)	V_OC_ (V)	FF	PCE (%)
1:1, w/w	7.96	0.707	0.27	1.50
1:2, w/w	9.03	0.730	0.30	2.00
1:3, w/w	9.68	0.792	0.35	2.69
1:4, w/w	9.95	0.735	0.31	2.23
